# A Multilingual Behavioral Speech Dataset for Vishing and Social Engineering Detection

**DOI:** 10.1038/s41597-026-07724-z

**Published:** 2026-06-29

**Authors:** Yasser Hmimou, Mohamed Tabaa, Azeddine Khiat, Zineb Hidila

**Affiliations:** 1 Multidisciplinary Laboratory of Research and Innovation (LPRI), Moroccan School of Engineering Sciences (EMSI), Casablanca, 20250 Morocco; 2 2IACS Laboratory, ENSET, Hassan II University of Casablanca, Casablanca, 20360 Morocco

## Abstract

Voice phishing (vishing) has emerged as a major vector for social engineering, exploiting the emotional and persuasive aspects of speech to deceive victims. Existing public datasets are often monolingual and lack structured annotations of persuasive intent and interactional structure in telephony scenarios. VISHGUARD is a multilingual synthetic audio corpus designed to advance research on persuasion-sensitive vishing detection. It contains 3,000 simulated phone calls in English, French, and Modern Standard Arabic evenly distributed between fraudulent and legitimate categories. Each sample is annotated across several dimensions, including persuasion strategy, interactional markers, and emotional tone. The audio recordings were synthetically generated using text-to-speech synthesis and controlled noise mixing, with ambient sounds used to approximate office, street, and home-like acoustic variability rather than full telecommunication-channel effects. The dataset covers durations from 10 to 90 seconds and maintains balanced linguistic and class distributions. VISHGUARD provides a reproducible, script-conditioned synthetic telephony corpus with multidimensional annotations to support controlled research on persuasion-aware vishing detection in multilingual settings. All data files are publicly available to ensure transparency and reuse.

## Background & Summary

Voice phishing, or vishing, has become a dominant vector for social engineering attacks that exploit the persuasive and emotional nuances of human language. By imitating credible authority, a sense of urgency, or empathy, adversaries manipulate their victims into revealing sensitive information or performing risky actions^1,2^, Unlike text-based phishing, vishing relies on the auditory channel, where tone, hesitations, and rhythm are commonly associated with persuasive speech strategies. The rise of advanced speech synthesis and voice cloning technologies has reduced the technical barriers to creating convincing synthetic voices^3,4^, leading to the global spread of AI-based phone scams that combine linguistic precision with emotional manipulation.

Over the past decade, research has focused primarily on acoustic authenticity detection, i.e., identifying whether a given signal is authentic or synthetic, rather than modeling behavioral deception. Public benchmarks such as ASVspoof 2019–2021 have enabled systematic evaluation of voice spoofing detection under controlled conditions. However, these resources mainly focus on short, monolingual, and emotionally neutral speech segments, which remain far from the conversational and psychological realism of vishing calls.

In parallel, deep learning architectures such as RawNet2^5^, ECAPA-TDNN^6^, and AASIST^7^ have driven substantial progress in spoofing detection by capturing low-level waveform inconsistencies and spectral artifacts left by neural vocoders. More recent approaches incorporate self-supervised representations such as wav2vec 2.0^8^ and cross-domain generalization techniques^9^, improving robustness across attack types and recording conditions.

Parallel developments in speech representation learning have advanced acoustic modeling and expressiveness analysis. Self-supervised paradigms such as wav2vec 2.0^10^ and HuBERT^11^ learn latent units directly from raw audio and have reshaped modern speech representation learning. Classic descriptors such as MFCCs^12^ laid the foundation for earlier statistical speech modeling approaches. More recent work, notably Prosody2Vec^13^, further emphasizes the role of intonation and rhythm in capturing emotional subtext.

In terms of data resources, widely used public corpora such as VoxCeleb^14^, LibriSpeech^15^, CommonVoice^16^, and SpeechFake^17^ continue to present speech primarily as an acoustic signal rather than an intentional act of persuasion. These datasets do not provide annotations for emotional polarity, behavioral patterns, or manipulation strategies, making them unsuitable for studying the progression of deceptive communication in vishing interactions.

At the same time, multimodal and LLM-based frameworks have introduced powerful cross-domain reasoning capabilities. Systems such as SpeechGPT^18^, LLaSM^19^, AudioGPT^20^, Qwen2-Audio^21^, and SeamlessM4T^22^ bridge linguistic and acoustic modalities, demonstrating that language models can interpret and even generate expressive speech. Yet, no publicly available dataset explicitly links linguistic content, prosodic behavior, and persuasive intent in a multilingual context. Existing studies on vishing detection, notably those by Shen *et al*.^3^ and Lee *et al*.^4^ and Park *et al*.^23^ rely primarily on binary labels (“fraudulent” or “legitimate”) without modeling the complex cues (tone, silence, or urgency) that make fraudulent speech persuasive.

Despite advances in spoofing detection and speech representation learning, publicly available resources still lack structured annotations explicitly linking persuasive intent to multilingual telephony scenarios. This limitation motivates the design of VISHGUARD as a controlled synthetic corpus focused on annotated vishing communication patterns, rather than on acoustic authenticity alone^15,24^.

The choice of English, French, and Arabic was motivated by both practical and scientific considerations. Vishing campaigns operate globally and frequently target multilingual populations, especially in regions where these three languages coexist in administrative, financial, and technical communication. English dominates international cybercrime infrastructures, French is widely used across Europe and Africa, and Arabic represents a major linguistic group in regions increasingly targeted by voice-based fraud.

From a methodological perspective, limiting the corpus to a single language would risk overfitting detection models to language-specific lexical or phonetic cues. By incorporating three typologically and phonetically distinct languages, VISHGUARD enables cross-linguistic robustness studies and reduces the bias toward monolingual acoustic patterns. This multilingual design supports controlled cross-linguistic evaluation and prototyping, while further real-world validation remains necessary for robust deployment-oriented detection systems.

To overcome these limitations, this paper presents VISHGUARD, a multilingual synthetic audio corpus focused on persuasion, specifically designed for vishing and social engineering detection. The dataset consists of a controlled collection of multilingual simulated calls, balanced across languages and interaction types, and designed to support reproducible benchmarking under constrained synthetic conditions. Unlike previous corpora, VISHGUARD does not contain any human-recorded voices, eliminating privacy concerns while preserving natural acoustic variability. Each sample is annotated along multiple behavioral and emotional dimensions to capture persuasive intent and interaction dynamics.

Compared to datasets such as ASVspoof (2019–2021), FakeAVCeleb^24^, or SpeechFake^17^, which mainly address acoustic spoofing, VISHGUARD focuses on script-conditioned vishing scenarios annotated with persuasive intent and interactional metadata under controlled synthetic conditions. Each entry is accompanied by a structured JSON file linking the audio clip to its transcript, language, and metadata, facilitating integration with automatic speech recognition, emotion recognition, and LLM-based reasoning systems. The dataset also provides a solid foundation for future multimodal studies linking vocal expression, linguistic framing, and the psychology of deception.

To clarify the positioning of VISHGUARD relative to existing public speech resources, Table 1 compares representative datasets in terms of scale, language coverage, speech origin, annotation type, availability, and target task. The comparison highlights that existing resources are primarily designed for speaker recognition, automatic speech recognition, spoofing detection, or speech deepfake detection, whereas VISHGUARD is designed for multilingual vishing-like scenarios with persuasion-pattern, perceived emotional-tone, and behavioral-marker annotations.

**Table 1:** Comparison of VISHGUARD with representative public speech datasets.

Dataset	Number of samples/scale	Languages	Speech origin	Main annotation types	Public availability	Target task
ASVspoof 2019–2021	Large challenge corpora; subset- and track-dependent	Mainly English	Real bonafide speech and spoofed or deepfake speech	Bonafide/spoof labels, attack conditions, challenge metadata	Public challenge data	Spoofing, replay, and deepfake speech detection
VoxCeleb/VoxCeleb2	More than 1 million utterances in VoxCeleb2	Multilingual and accent-diverse speech from online videos	Real human speech	Speaker identity and utterance/video metadata	Public or access-controlled depending on version and terms of use	Speaker recognition and speaker verification
LibriSpeech	Approximately 1,000 hours of read speech	English	Real read audiobook speech	Orthographic transcripts	Public	Automatic speech recognition
Common Voice	Version-dependent large-scale crowdsourced corpus	Multilingual	Real crowdsourced speech	Transcripts and selected speaker/session metadata depending on release	Public	ASR and multilingual speech technology
SpeechFake	More than 3 million synthetic/deepfake samples and more than 3,000 hours	46 languages	Synthetic/deepfake speech generated using multiple speech-generation tools	Real/fake or generation-related labels	Public according to the dataset release	Speech deepfake detection
VISHGUARD	3,000 calls, approximately 32 hours	English, French, Modern Standard Arabic	Script-conditioned synthetic TTS speech with controlled ambient-noise mixing	Class label, transcript, persuasion patterns, perceived emotional tone, behavioral markers	Public Zenodo record	Vishing and social-engineering detection

By combining voice forensics, structured annotation, and AI-based cybersecurity, VISHGUARD provides a reproducible multilingual resource for analyzing persuasive vocal manipulation. Recent efforts have also explored compact AI architectures for efficient vishing detection across low-resource environments^25^. It supports research at the intersection of speech processing and cybersecurity by providing structured multilingual scripts aligned with annotated persuasion patterns and interactional cues, without claiming that synthesized prosody constitutes human behavioral ground truth. All data and generation scripts are made public to promote transparency, reproducibility, and interdisciplinary collaboration in speech-based cybersecurity research.

### Scope and limitations of the synthetic design

VISHGUARD should be interpreted as a controlled, script-conditioned synthetic corpus for studying multilingual vishing-like communication patterns, rather than as a direct substitute for real-world vishing recordings. Because all voices were generated using text-to-speech synthesis, the corpus does not capture the full range of spontaneous human vocal behaviour, cognitive load, hesitation, speaker adaptation, emotional pressure, or deception-related cues that may occur in real victim-attacker interactions. The emotional and interactional annotations should therefore be understood as perceived communicative metadata derived from scripted scenarios and synthesized audio, not as measurements of human psychological states or real deception behaviour. The dataset is most appropriate for benchmarking, prototyping, controlled comparative studies, and the development of detection pipelines that combine transcript-level and audio-level evidence. Performance obtained on VISHGUARD should not be interpreted as evidence of direct deployment readiness without additional validation on real or naturally collected telephony data.

## Methods

**Fig. 1:**
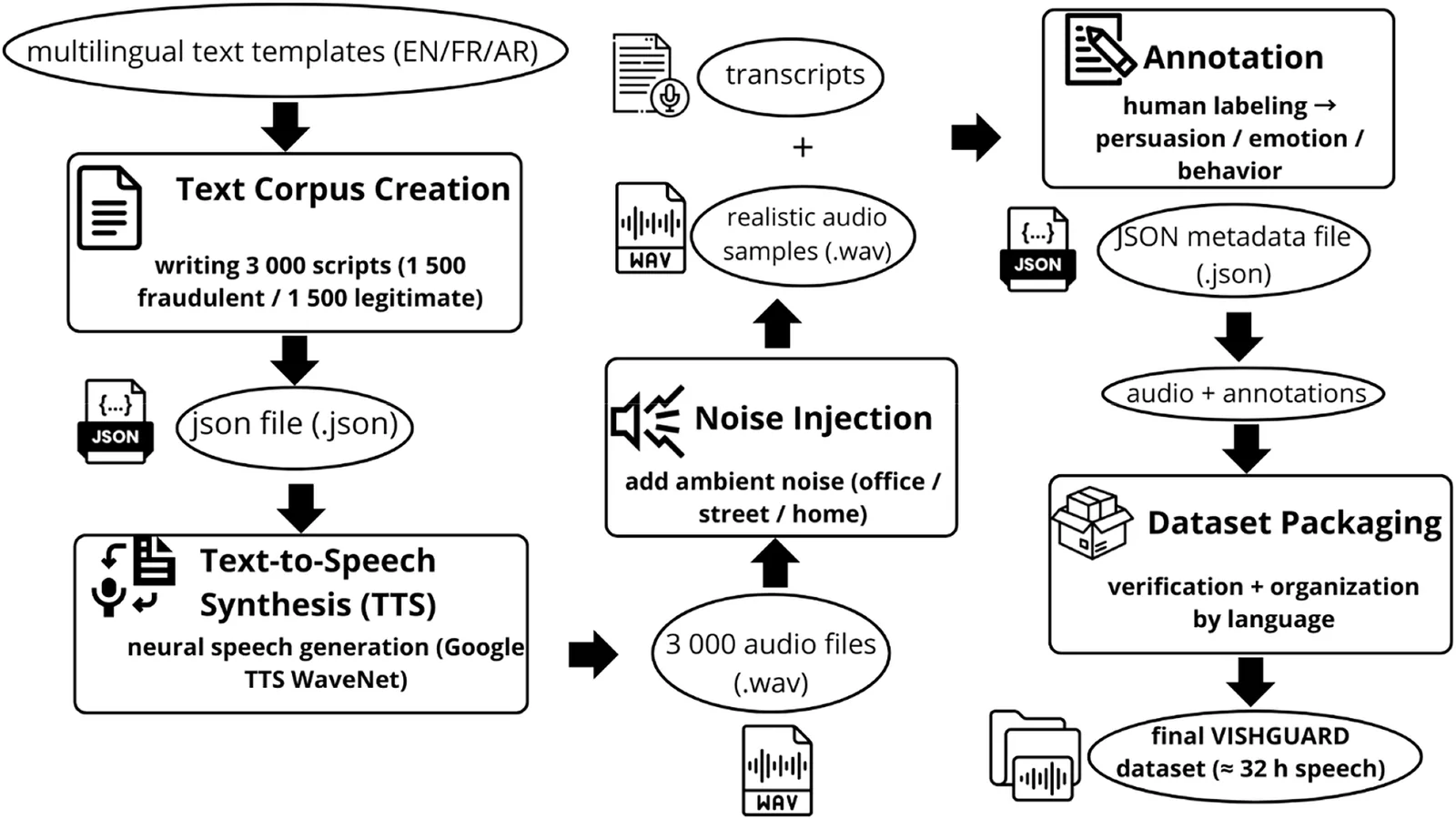
Simplified overview of the VISHGUARD dataset generation pipeline.

The VISHGUARD corpus was designed as a controlled but realistic simulation of voice phishing and legitimate interactions. Its construction follows a reproducible process comprising (i) multilingual speech synthesis generation, (ii) acoustic realism enhancement via noise composition, and (iii) multidimensional behavioral annotation. As shown in Fig. 1, each step has been validated to ensure linguistic diversity, acoustic consistency, and annotation reliability.

### Audio generation pipeline

To simulate authentic telecommunications conditions, all voice samples were synthetically generated using Google Cloud Text-to-Speech (TTS), which relies on neural speech synthesis architectures inspired by WaveNet-based waveform generation models^26^ and Tacotron-style acoustic conditioning frameworks^27^. Neural voices were selected by language (en-US-Wavenet, fr-FR-Neural2, and ar-XA-Wavenet models). A single neural voice model was used per language to maintain consistency across samples. The TTS phase produced semantically consistent scripts representing two categories: fraudulent calls (vishing) and legitimate (non-fraudulent) calls. The fraudulent scripts were inspired by social engineering strategies documented in financial, administrative, and technical scams^3,28,29^, while the legitimate scripts reproduced neutral inquiries or service calls. All scripts were originally written by the authors, and no external speech or text datasets were used as input data for corpus construction. Ambient background sounds were used as environmental noise sources during the mixing process. For each third-party sound source, the licensing conditions were checked and documented in a license manifest accompanying the dataset documentation. To avoid redistribution of third-party audio as standalone material, unmodified ambient sound files are not distributed as separate audio resources in the released dataset. Instead, ambient signals are incorporated into the final synthetic call recordings through controlled noise mixing.

Speech synthesis was performed using the Google Cloud Text-to-Speech REST API (endpoint: https://texttospeech.googleapis.com/v1/text:synthesize) A total of 3,000 independent POST requests were sent to the endpoint, one per script. All requests were authenticated through a paid Google Cloud account using an API key. For each script, a JSON payload containing the full transcript and selected neural voice model was submitted individually.

The following synthesis parameters were used consistently across all samples:

– audioEncoding: LINEAR16

– sampleRateHertz: 16000

– speakingRate: 1.0

– pitch: 0.0

– volumeGainDb: 0.0

The returned Base64-encoded audio content was decoded and stored as 16-bit PCM WAV files without additional pitch or tempo modification.

All calls were synthesized using a single predefined neural voice per language, with identical synthesis parameters applied across the entire corpus. This choice was made to maintain synthesis consistency and reduce uncontrolled variation during the first release of the dataset. However, it also limits speaker diversity and may cause models trained only on VISHGUARD to learn voice-specific or TTS-engine-specific characteristics. The current version should therefore not be used to draw conclusions about robustness across speakers, genders, accents, or speaking styles without additional validation. No SSML-based prosodic manipulation, style tokens, expressive controls, or emotion-conditioning parameters were introduced.

Specifically:

– no explicit pause-duration control or tempo variation was manually applied.

Pauses and temporal structure emerge solely from punctuation and textual organization within the scripts as interpreted by the neural TTS engine.

No explicit modeling of cognitive load, emotional state, or human deception mechanisms was introduced during synthesis. Acoustic variability therefore results from the internal text-to-prosody mapping of the neural TTS system conditioned by the scripts, and should not be interpreted as behavioral ground truth.

To avoid repetition and improve acoustic realism, layers of ambient noise were added from a selection of ambient sounds, including office conversations, traffic, and household background noise. The inclusion of diverse ambient conditions (office, street, and home environments) does not imply that attackers necessarily operate from these specific locations. Instead, these noise profiles were selected to approximate realistic telecommunication scenarios, including mobile phone usage, VoIP calls, and background environmental variability commonly observed in real-world conversations. Empirical studies on telephony-based fraud indicate that vishing calls may originate from heterogeneous acoustic conditions due to the widespread use of mobile devices, call-centers, and VoIP infrastructures. Incorporating multiple ambient profiles therefore enhances acoustic robustness and ecological validity, ensuring that detection models trained on VISHGUARD do not overfit to artificially clean speech conditions.

Each audio file was mixed at a random signal-to-noise ratio (SNR) between 15 and 25 dB to maintain intelligibility while imitating the variability of real-world calls. The final corpus totals 3,000 recordings, evenly split between 1,500 legitimate samples and 1,500 fraudulent samples, representing approximately 32 hours of conversation. The duration varies from 10 to 90 seconds (average 33 s). All audio files are stored in 16 kHz, 16-bit mono PCM format, in accordance with telephony standards. Noise mixing was implemented using Python (librosa version 0.10.1). For each sample, the selected ambient noise waveform was randomly cropped to match the speech duration. The SNR value was uniformly sampled between 15 dB and 25 dB. Noise amplitude scaling followed the standard signal power formula:

$${\rm{noise\_scaled}}={\rm{noise}}\times {\rm{sqrt}}\left({\rm{P\_speech}}/\left({{\rm{P}}}_{{\rm{noise}}}\times {10}^{\left({\rm{SNR}}/10\right)}\right)\right)$$

where P_speech and P_noise denote signal power. The final waveform was normalized to prevent clipping before export.

#### Telephony-channel limitations

The present release simulates telephony-like acoustic variability through controlled ambient-noise mixing at 15–25 dB SNR and stores all recordings as 16 kHz, 16-bit mono PCM WAV files. However, it does not explicitly model the full range of real telecommunication-channel effects, such as narrowband or wideband codec compression, packet loss, jitter, reverberation, device-specific microphone responses, bandwidth limitation, automatic gain control, or VoIP transmission artefacts. These factors may influence the performance of vishing-detection systems in operational settings. Future versions of VISHGUARD should include explicit codec simulation, narrowband and wideband telephony transformations, packet-loss modelling, reverberation profiles, and microphone/channel variability to better approximate real phone-call conditions.

### Corpus Structure and language distribution

VISHGUARD adopts multilingual coverage (40% in English, 30% in French, and 30% in Arabic). Each subset maintains identical proportions of fraudulent and legitimate examples, ensuring a balance between languages and acoustic profiles. For the Arabic subset, Modern Standard Arabic (MSA) was used rather than dialectal Arabic. This choice was motivated by the need for linguistic consistency, cross-regional comprehensibility, and compatibility with neural text-to-speech models. MSA is widely employed in formal administrative, financial, and institutional communication, which aligns with the typical framing of vishing scenarios (e.g., bank notifications, government warnings, technical support calls).

While dialectal Arabic may better reflect informal daily conversations, its high intra-regional variability could introduce uncontrolled lexical and phonetic heterogeneity. The use of MSA therefore ensures reproducibility, linguistic standardization, and better cross-linguistic comparability within the dataset. Nevertheless, this design choice limits direct applicability to Arabic-speaking regions where real vishing calls may rely on dialectal Arabic, informal spoken varieties, or code-switching between Arabic, French, and English. The Arabic subset should therefore be interpreted as a standardized MSA component rather than a comprehensive representation of Arabic vishing speech across dialectal communities.

Each sample is uniquely indexed and accompanied by a structured JSON metadata file describing:

- id: unique identifier,
- language: ISO 639-1 code (en, fr, ar),
- class: “fraudulent” or “legitimate,”
- audio_path: relative file path,
- transcript: textual content,
- noise_type: ambient source,
- duration: in seconds.

### Annotation protocol and behavioral dimensions

Each audio sample was annotated manually. Annotators labeled each sample using five predefined persuasion categories: authority, scarcity, urgency, reward, and threat. At the same time, emotional tone was classified into several categories: neutral, manipulative, anxious, calm, or authoritative, while behavioral cues such as long silences, interruptions, and irregular speech rate were noted in order to capture the interactional dynamics.

All annotations were consolidated by majority vote, and agreement among annotators reached a Cohen’s κ coefficient of 0.87, reflecting high reliability. Three independent annotators labeled each sample following a predefined annotation guideline document included in the dataset documentation deposited with the Zenodo record. Annotation was performed independently in a first phase and followed by consensus review. Cohen’s kappa coefficient was computed using scikit-learn (version 1.3) to quantify inter-annotator agreement.

### Annotator background, training, and disagreement resolution

The annotation was carried out by three independent annotators with experience in multilingual communication, speech-data analysis, and cybersecurity-related content review. Before the main annotation phase, annotators received a written annotation guideline defining each persuasion category, emotional-tone category, and behavioral marker. The guideline specified label definitions, inclusion and exclusion criteria, examples of typical and borderline cases, and decision rules for multi-label persuasion annotations.

A calibration phase was conducted on an initial subset of samples to harmonize the interpretation of the annotation categories before full labeling. During the main annotation phase, annotators worked independently and were provided with both the synthesized audio file and the aligned transcript. After independent labeling, annotations were consolidated by majority vote. Cases without clear agreement or with ambiguous label interpretation were reviewed during a consensus phase, during which the final label was assigned after discussion based on the written guideline. The annotation guideline and representative examples are provided in the accompanying dataset documentation deposited with the Zenodo record.

Annotators were provided with both the synthesized audio file and its aligned transcript during the labeling process. The annotation layers were separated according to the type of evidence used during labeling. Persuasion-pattern labels were primarily script- and transcript-based, because categories such as authority, scarcity, urgency, reward, and threat are defined by communicative intent and lexical framing. Emotional-tone labels were assigned using both the transcript and the synthesized audio, reflecting perceived communicative stance rather than physiological emotion. Behavioral markers, such as long silences, interruptions, and irregular speech rate, were assigned from observable properties of the generated waveform and audio realization, including pause duration, restart timing, and rhythm variability.

This separation is important because not all annotation layers should be interpreted as purely acoustic labels. The persuasion-pattern annotations characterize communicative intent encoded in the script, while the behavioral markers describe observable interactional properties of the synthesized audio. This design enables both transcript-based and audio-based modelling, but it also requires users to select annotation targets according to the modality used in their experiments.

Importantly, these behavioral labels do not represent inferred psychological states, but rather structured descriptors of interactional patterns present in the synthesized signal. Scripts did not contain explicit behavioral tags or pause instructions; therefore, annotators relied on the final audio realization rather than predefined script-level cues.

Annotations are stored in parallel JSON files linked to their respective audio samples, ensuring traceability and easy integration into machine learning pipelines.

**Fig. 2:**
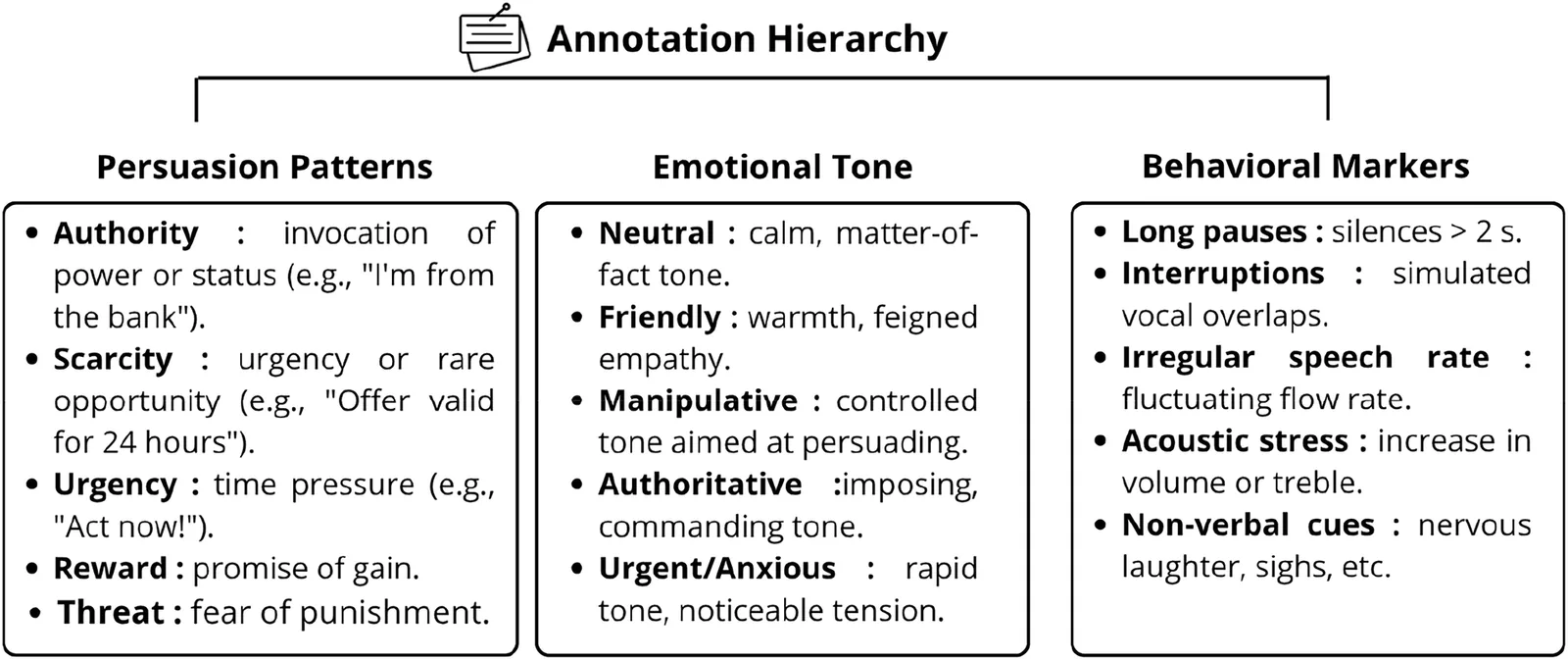
Hierarchical annotation taxonomy of VISHGUARD.

Figure 2 shows the three levels of labeling: persuasion strategies, emotional tone, and behavioral markers.

#### Persuasion patterns

Persuasive intent is a characteristic attribute of fraudulent communication. Based on the Cialdini influence model, each fraudulent sample was analyzed to identify one or more of the following five persuasion strategies: authority, scarcity, urgency, reward, and threat. These dimensions reflect the recurring manipulation tactics observed in financial, administrative, and identity theft scams^3,28^.

Each strategy is stored as a boolean field in the JSON schema (persuasion_authority, persuasion_threat, etc.), allowing for multi-label annotation when multiple techniques coexist. To clarify the distinction between fraudulent and legitimate samples, the two classes differ primarily in communicative intent and persuasive framing. Fraudulent calls typically include labelled persuasive triggers such as urgency, authority, reward, scarcity, or threat, while legitimate calls generally reproduce service, administrative, or customer-support interactions without fraudulent intent.

This controlled script-level contrast supports reproducible annotation and benchmarking. However, it may also make within-corpus classification easier than real-world vishing detection, where legitimate calls can contain urgency, authority, or administrative pressure without being fraudulent, and fraudulent calls can be indirect, low-pressure, or ambiguous. Future versions of VISHGUARD should therefore include harder legitimate examples containing urgency or authority without fraud, as well as subtler fraudulent examples that do not rely on explicit threats or obvious pressure cues.

Behavioral and interactional markers are provided as structured annotations to facilitate downstream analysis; however, no claim is made that synthesized prosodic realizations correspond to human cognitive or deception mechanisms.

#### Emotional tone encoding

Vocal emotions strongly influence the effectiveness of persuasion. To capture this dimension, each statement was classified according to five affective categories: neutral, manipulative, calm, anxious, and authoritative.

The emotional tone categories reflect perceived communicative stance in the scripted scenarios (e.g., manipulative, anxious, authoritative). These labels are assigned through manual annotation and represent categorical metadata for downstream modeling, without implying direct measurement of physiological or cognitive speech-production processes.

Each emotional label was assigned by agreement among annotators, who assessed valence and arousal levels using a 3-point Likert scale, then grouped into categorical values.

#### Behavioral markers

Beyond persuasion and emotion, temporal and interactional markers (e.g., long silences, interruptions, irregular rhythm) are annotated to provide structured metadata for research on vishing communication patterns in a controlled synthetic setting^30,31^. Annotators identified three temporal and acoustic markers: long silences (pauses >800 ms), interruptions (overlaps or restarts within 300 ms), and irregular prosodic rhythm (unstable pitch contour or non-linear tempo).

All markers are encoded as digital metadata in the corpus, allowing for continuous or categorical modeling.

### Dataset construction timeline

The full construction process of the VISHGUARD dataset was conducted over a period of approximately four months. Script design and multilingual preparation required six weeks, followed by two weeks of automated text-to-speech synthesis and acoustic post-processing (including noise injection and quality verification). The manual annotation phase was completed over five weeks by three independent annotators, including independent labeling and consensus review sessions. Final validation, schema consistency checks, and packaging for public release required an additional three weeks. This timeline reflects the controlled and versioned workflow adopted to ensure reproducibility and annotation reliability.

### Ethics statement

All voices in the VISHGUARD corpus were synthetically generated using text-to-speech technology. No human participants were recorded, and no personal or sensitive information is included. Consequently, no ethical approval or consent procedures were required.

Because the dataset contains synthetic vishing-like scenarios, we also considered potential dual-use risks before public release. The dataset is intended exclusively for defensive cybersecurity research, detection benchmarking, awareness, and educational analysis. To reduce harmful reuse, the released scripts and examples were de-operationalized and do not include real institutional names, real phone numbers, URLs, account identifiers, payment details, credential-collection instructions, telephony-deployment tools, or targeted victim-profiling functionality. Additional safeguards are described in the Misuse-risk assessment and safeguards subsection.

## Data Records

The dataset is available at Zenodo^32^. It consists of structured directories containing the final mixed audio recordings, aligned transcripts, JSON-based annotation metadata, validation files, and documentation. Third-party ambient noise sources used during generation are documented in a license manifest but are not redistributed as unmodified standalone audio files. The complete dataset occupies approximately 6.5 GB of storage space and contains 3,000 final mixed WAV files (16 kHz, 16-bit PCM), aligned transcripts, JSON-based annotations, metadata, and accompanying documentation.

The documentation folder also includes the annotation guideline document used during labeling, together with representative label definitions and examples.

The scripts folder contains generation, controlled noise-mixing, metadata-validation, audio-quality-control, annotation-agreement, packaging, and baseline-validation scripts used to support reproducibility.

**Fig. 3:**
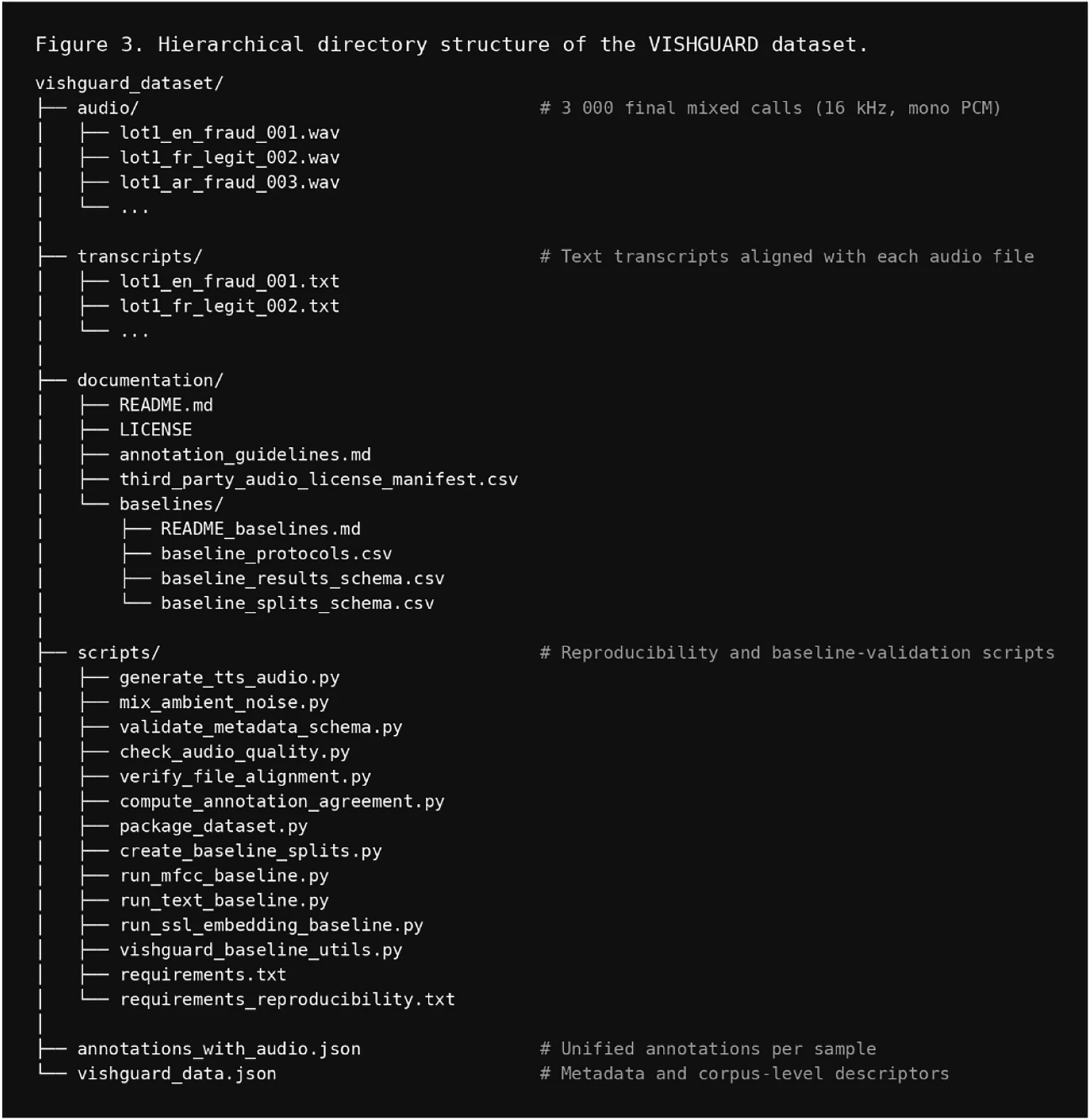
Hierarchical directory structure of the VISHGUARD dataset, including final mixed audio files, aligned transcripts, JSON annotation metadata, corpus-level metadata, documentation files, baseline-validation files, and reproducibility scripts deposited with the Zenodo record. Third-party ambient noise sources used during generation are documented in a license manifest but are not redistributed as standalone audio resources.

The overall directory organization of the dataset is illustrated in Fig. 3.

### Audio files

Each.wav file represents a single simulated call aligned with its transcript and metadata entry. Detailed audio specifications (sampling rate, encoding format, and duration range) are provided in the Methods section.

File names follow the pattern:

lot[batch_id]_[language]_[type]_[id].wav

- language ∈ {en, fr, ar}
- type ∈ {fraud, legit}
- id is a numeric identifier whose range depends on the language subset (e.g., 001–1200 for English and 001–900 for French and Arabic).

### Text transcripts

For every audio file, a plain-text transcript provides the full spoken content used during synthesis. Scripts include realistic human phrasing and limited code-switching phenomena. Code-switching, when present, occurs at the lexical or short phrase level (e.g., insertion of English technical terms within French or Arabic sentences) and remains intra-speaker within a single TTS voice configuration. No dynamic voice switching or multi-speaker synthesis was performed within a single audio file.

### Annotation files

Each record in annotations_with_audio.json links linguistic, emotional, and behavioral layers

{“id”: “lot1_en_fraud_001”,

“type”: “fraudulent”,

“language”: “en”,

“duration”: 48.0,

“emotion”: [“anxious”, “authoritative”],

“persuasion_patterns”: [“threat”, “urgency”, “authority”],

“behavior”: [“irregular_speech”, “interruptions”],

“metadata”: {“words_count”: 115,

“audio_path”: “audio/lot1/en_fraud_001.wav”}}

The annotation fields differ in their evidential basis. The ‘persuasion_patterns’ field is primarily script/transcript-based, the ‘emotion’ field is based on perceived communicative stance from both transcript and synthesized audio, and the ‘behavior’ field is based on observable properties of the generated audio signal. Users should therefore interpret each annotation layer according to its intended modality.

The JSON annotation schema is summarized in Table 2. Each field has a defined schema:

**Table 2:** Description of JSON Metadata Fields in the VISHGUARD Annotation Files.

Field	Description
id	Unique identifier combining batch, language, and call type
type	Category label — fraudulent or legitimate
language	ISO 639-1 code (en, fr, ar)
duration	Duration in seconds
emotion	Perceived communicative tone based on transcript and synthesized audio
persuasion_patterns	Script/transcript-based persuasion triggers (authority, scarcity, reward, threat, urgency)
behavior	Audio-based interactional cues observed in the generated waveform (interruptions, irregular speech, long silence)
metadata	Nested object with word count and relative path to audio file

## Data Overview

Table 3 provides a detailed statistical overview of the dataset composition.

**Table 3:** Statistical Overview of the VISHGUARD Dataset Composition.

Language	Fraudulent Samples	Legitimate Samples	Total Samples	Average Duration (s)	Duration Range (s)	Proportion (%)
English	600	600	1 200	32.4	10–90	40
French	450	450	900	31.8	10–85	30
Arabic (MSA)	450	450	900	33.7	12–88	30
Total	1 500	1 500	3 000	32.6 (μ)	10–90	100%

## Technical Validation

The dataset underwent multiple structural, statistical, and acoustic quality-control procedures to ensure integrity and reproducibility.

Language distribution was verified to remain within ±2% of the intended proportions (40% English, 30% French, 30% Arabic), and class parity was exact (1,500 fraudulent and 1,500 legitimate samples).

Audio files were automatically checked for format consistency (16 kHz, 16-bit PCM mono), duration range (10–90 seconds), and absence of clipping after noise injection. Acoustic diversity was assessed using short-time energy and spectral flatness metrics to confirm variability across ambient noise conditions while preserving intelligibility.

Annotation reliability was evaluated using Cohen’s kappa coefficient computed with scikit-learn (v1.3). Agreement reached κ = 0.87 for persuasion patterns, κ = 0.84 for emotional tone, and κ = 0.81 for behavioral markers, indicating substantial inter-annotator consistency.

All annotation files were version-controlled prior to final release, and schema consistency checks were performed to ensure alignment between audio files, transcripts, and metadata entries.

### Baseline-validation protocols

To support reproducible reuse, we deposited simple baseline-validation protocols and scripts with the Zenodo record. These materials are intended to verify that VISHGUARD can support standard audio-based, transcript-based, and representation-learning workflows, rather than to establish state-of-the-art performance.

The Zenodo record includes baseline scripts, protocol files, and schema files for reproducible evaluation using stratified train, validation, and test splits that preserve language and class balance. Three baseline settings are provided: (i) MFCC-based audio classification using logistic regression, (ii) self-supervised speech representations extracted from wav2vec 2.0 or HuBERT followed by logistic regression, and (iii) transcript-based classification using multilingual TF-IDF text features. The scripts are designed to report accuracy and macro-F1 when executed.

These baseline materials should be interpreted as reproducibility and usability resources rather than as a state-of-the-art benchmark. Because the present release is synthetic and intentionally structured at the script level, external validation on real or naturally collected telephony data remains necessary before operational use.

### Misuse-risk assessment and safeguards

Because VISHGUARD contains synthetic vishing-like scenarios, the released materials were reviewed for potential misuse before public deposition. The dataset is intended exclusively for defensive cybersecurity research, detection benchmarking, awareness, and educational analysis. To reduce harmful reuse, the scripts were de-operationalized: they do not contain real institutional names, real phone numbers, URLs, account identifiers, payment details, credential-collection instructions, or procedural guidance for conducting fraud. Fraudulent examples are provided as generic, labelled detection samples rather than reusable scam templates. No human voice recordings, speaker identities, or voice-cloning material are included. The released code and documentation are limited to dataset validation, metadata consistency checks, audio-quality verification, noise-mixing documentation, and reproducible packaging. They do not provide tools for telephony deployment, automated calling, targeted victim profiling, or persuasion optimization. The dataset documentation explicitly frames VISHGUARD for defensive detection research and prohibits harmful use. These safeguards reduce potential misuse while preserving the dataset’s value for reproducible research on vishing detection.

## Usage Notes

VISHGUARD is distributed in open formats (.wav,.txt, and.json) to facilitate reuse with common audio-processing and machine-learning frameworks, including Python-based workflows, PyTorch, and TensorFlow. Each sample can be linked across the audio, transcript, and annotation files using the unique sample identifier.

The dataset can support several defensive research tasks, including binary vishing detection, multilingual speech classification, transcript-based persuasion-pattern analysis, audio-text modelling, and robustness evaluation under controlled ambient-noise conditions.

Although VISHGUARD contains 3,000 samples and approximately 32 hours of audio, it should not be interpreted as providing broad coverage of all real-world vishing conditions or as sufficient by itself for training robust multilingual deep learning systems from scratch. The dataset is intended primarily for controlled benchmarking, prototyping, annotation-aware modelling, pipeline development, and pretraining or evaluation support. Robust multilingual deployment would require additional data covering more speakers, dialects, spontaneous interactions, telephony channels, recording devices, and naturally occurring call conditions.

Users should consider the controlled nature of the corpus when designing experiments. All voices are synthetically generated, one predefined neural voice model was used per language, and the corpus does not contain spontaneous human conversations or real victim-attacker interactions. As a result, VISHGUARD does not fully capture human hesitation, cognitive load, emotional pressure, speaker adaptation, or deception-related vocal behaviour observed in real calls. The emotional and interactional labels should therefore be interpreted as perceived communicative and annotation metadata rather than measurements of human psychological states or deception behaviour. Models trained or evaluated on VISHGUARD should be further validated on real or naturally collected telephony data before any operational deployment.

The single-voice-per-language design is a major limitation of the current release. Although it improves internal consistency, it reduces speaker diversity and may increase the risk that classifiers learn voice-specific, language-specific, or TTS-engine-specific artefacts rather than generalizable vishing-related cues. Future versions of VISHGUARD should include multiple neural voices per language, male and female voices, regional accents, different speaking rates, expressive styles, and outputs from multiple TTS systems to better support speaker-independent and cross-domain evaluation.

The current release should also not be interpreted as a complete simulation of real telecommunication channels. Although ambient noise was added under controlled SNR conditions, the recordings do not include explicit codec compression, packet loss, jitter, reverberation, automatic gain control, device-specific microphone responses, or VoIP artefacts. Users evaluating operational vishing-detection systems should therefore test models on additional data containing real or simulated telephony-channel effects.

The Arabic subset uses Modern Standard Arabic to maintain linguistic consistency, cross-regional comprehensibility, and compatibility with available neural TTS systems. However, this choice limits real-world applicability in Arabic-speaking regions where vishing calls may involve dialectal Arabic, informal registers, or code-switching with French or English. Future versions of VISHGUARD should include dialectal Arabic variants, such as Moroccan Arabic, Egyptian Arabic, Gulf Arabic, and Levantine Arabic, to better reflect regional communication practices and improve ecological validity.

The dataset is intended for defensive cybersecurity research, benchmarking, awareness, and educational use. It should not be used to develop, optimize, deploy, or automate fraudulent communication systems.

The fraudulent and legitimate classes in the current release are intentionally separated at the script level to support controlled benchmarking and reproducible annotation. This structure may make within-corpus classification easier than detection in real operational settings, where legitimate calls may contain urgency, institutional authority, or administrative pressure without fraud, and fraudulent calls may be subtle, indirect, low-pressure, or ambiguous. Users should therefore interpret classification performance on VISHGUARD as controlled benchmark performance rather than evidence of real-world operational robustness.

For reproducible reuse, the Zenodo record also provides baseline scripts, protocol files, and schema files for creating stratified splits and running MFCC-based, self-supervised speech, and transcript-based baseline protocols.

## Data Availability

The VISHGUARD dataset is available at Zenodo: https://doi.org/10.5281/zenodo.20429763. The Zenodo record contains the complete final mixed audio corpus (.wav), transcripts (.txt), annotation files (.json), metadata, documentation, supporting scripts, and a third-party audio license manifest. Unmodified third-party ambient sound files are not redistributed as standalone audio resources.

## Code availability

No installable standalone software package is required to access or use the dataset. To support reproducibility, the Zenodo record includes Python scripts for dataset generation, audio post-processing, metadata validation, audio-quality control, annotation-agreement computation, baseline-validation protocols, and dataset packaging: https://doi.org/10.5281/zenodo.20429763. The deposited scripts include: generate_tts_audio.py for reproducing the text-to-speech request workflow, mix_ambient_noise.py for controlled ambient-noise mixing, validate_metadata_schema.py for checking JSON metadata fields, check_audio_quality.py for verifying sampling rate, channel count, duration range, and clipping, verify_file_alignment.py for checking alignment between audio files, transcripts, and annotation records, compute_annotation_agreement.py for reproducing Cohen’s kappa calculations, and package_dataset.py for recreating the released directory structure. The Zenodo record also includes baseline-validation scripts for MFCC-based audio classification, self-supervised speech embedding extraction, and transcript-based text classification.

Google Cloud authentication credentials, API keys, and account-specific configuration files are not included for security reasons. Users wishing to reproduce the TTS generation workflow must provide their own Google Cloud credentials and comply with the relevant service terms. No proprietary or closed-source algorithm is required to access, validate, or use the released dataset.
